# Cyanobacterial Polyhydroxybutyrate (PHB): Screening, Optimization and Characterization

**DOI:** 10.1371/journal.pone.0158168

**Published:** 2016-06-30

**Authors:** Sabbir Ansari, Tasneem Fatma

**Affiliations:** Cyanobacterial Biotechnology Laboratory, Department of Biosciences, Jamia Millia Islamia (Central University), New Delhi, India; Laurentian University, CANADA

## Abstract

In modern life petroleum-based plastic has become indispensable due to its frequent use as an easily available and a low cost packaging and moulding material. However, its rapidly growing use is causing aquatic and terrestrial pollution. Under these circumstances, research and development for biodegradable plastic (bioplastics) is inevitable. Polyhydroxybutyrate (PHB), a type of microbial polyester that accumulates as a carbon/energy storage material in various microorganisms can be a good alternative. In this study, 23 cyanobacterial strains (15 heterocystous and 8 non-heterocystous) were screened for PHB production. The highest PHB (6.44% w/w of dry cells) was detected in *Nostoc muscorum* NCCU- 442 and the lowest in *Spirulina platensis* NCCU-S5 (0.51% w/w of dry cells), whereas no PHB was found in *Cylindrospermum sp*., *Oscillatoria sp*. and *Plectonema sp*. Presence of PHB granules in *Nostoc muscorum* NCCU- 442 was confirmed microscopically with Sudan black B and Nile red A staining. Pretreatment of biomass with methanol: acetone: water: dimethylformamide [40: 40: 18: 2 (MAD-I)] with 2 h magnetic bar stirring followed by 30 h continuous chloroform soxhlet extraction acted as optimal extraction conditions. Optimized physicochemical conditions viz. 7.5 pH, 30°C temperature, 10:14 h light:dark periods with 0.4% glucose (as additional carbon source), 1.0 gl^-1^ sodium chloride and phosphorus deficiency yielded 26.37% PHB on 7^th^ day instead of 21^st^ day. Using FTIR, ^1^H NMR and GC-MS, extracted polymer was identified as PHB. Thermal properties (melting temperature, decomposition temperatures etc.) of the extracted polymer were determined by TGA and DSC. Further, the polymer showed good tensile strength and young’s modulus with a low extension to break ratio comparable to petrochemical plastic. Biodegradability potential tested as weight loss percentage showed efficient degradation (24.58%) of PHB within 60 days by mixed microbial culture in comparison to petrochemical plastic.

## Introduction

Bioplastic can be defined as a plastic derived from renewable biological materials, which excludes the biomass embedded in geological formation or transformed into fossil fuels. Bioplastics produced from renewable carbon resources add to our efforts to conserve finite fossil resources, like mineral oil and coal, which are directly or indirectly used for plastic production. Degradation of bioplastics takes much lesser time as compared to petroleum-based plastics. The degradation products of bioplastics are carbon dioxide and water [1]. Bioplastic can also be implanted in the body without causing inflammation. Their biocompatibility is making them innovative products in the medical field. Some possible bioplastics applications include biodegradable carriers, surgical needles, suture materials, bone tissue replacement materials, etc [2,3].

PHB is a common biopolymer which is an attractive alternative to common plastics due to its hydrophobicity, complete biodegradability and biocompatibility [4]. Many gram-positive and gram-negative bacteria (*Pseudomonas* sp., *Bacillus* sp., *Methylobacterium* sp.) synthesize PHB [5]. However, cyanobacteria are the only oxygen-producing photosynthetic prokaryotes that accumulate PHB. Since the discovery of PHB in the cyanobacterium, *Chloroglea fritschii* [6], the occurrence of PHB has been shown in many cyanobacterial species [7–9]. So far low PHB content (<10% dcw) has been reported in cyanobacteria under photoautotrophy. Looking into the demand of more and more bioplastic to replace nonbiodegradable petroleum derived plastics, there is an urgent need of thorough screening of biodiversity, optimization of culture conditions, processing steps and characterization for PHB. In the present study, the potential of 23 cyanobacterial strains was screened for PHB production. The best strain, *Nostoc muscorum* NCCU- 442 was selected for optimization of PHB extraction, physiochemical culture conditions and time course study. Fourier Transform Infrared spectroscopy (FTIR), proton Nuclear Magnetic Resonance **(**^1^H NMR), Gas Chromatography- Mass Spectrometry (GC-MS), were used for chemical characterization whereas Thermogravimetric analysis (TGA) and Differential Scanning Calorimetry (DSC) were utilised for thermal characterization of PHB. Mechanical properties (tensile strength, young’s modulus and elongation to break ratio) and biodegradation potentiality of the PHB film formed after solvent casting method were also determined.

## Materials and Methods

### Screening of cyanobacteria for PHB

Twenty three cyanobacterial strains (fifteen heterocystous and eight non-heterocystous) were procured from National Centre for Collection and Utilisation of Blue Green Algae, Indian Agricultural Research Institute, New Delhi-110012. The heterocystous cyanobacterial strains were *Anabaena sp*. NCCU-9, *Anabaena variabilis* NCCU-441, *Aphanocapsa* NCCU-542, *Aulosira fertilissma* NCCU-443, *Calothrix brevissima* NCCU-65, *Cylindrospermum sp*. NCCU-272, *Hapalosiphon fontinalis* NCCU-339, *Michrochaete sp*. NCCU-342, *Nostoc punctiforme*, *Nostoc paludosum* NCCU-63, *Nostoc muscorum* NCCU- 442, *Nostoc sphaericum*, *Scytonema sp*. NCCU-126, *Tolypothrix tenuis* NCCU-122, and *Westiellopsis prolifica* NCCU-331, whereas the non-heterocystous strains were *Chrococcus sp*. NCCU-207, *Gleocapsa gelatinosa* NCCU-430, *Lyngbya sp*. NCCU-102, *Oscillatoria sp*. NCCU-369, *Phormidium sp*. NCCU-104, *Plectonema sp*. NCCU-204, *Spirulina platensis* NCCU-S5 and *Synechocystis sp*. NCCU-370.

Axenic cultures of cyanobacterial strains were maintained in a temperature-controlled incubator at 28 ± 1°C under a photoperiod of 14:10 h (light: dark cycle) at 25 μmol photon m^-2^ s^-1^. The non-heterocystous forms were cultured in BG-11 with NaNO_3_ and the heterocystous forms in NaNO_3_ free BG-11 medium [10]. *Spirulina platensis* was cultivated in Zarruk’s medium [11]. Shaking was done at regular intervals for mixing of nutrients and aeration. Twenty one days old biomass was filter-harvested, using Whatman no.1 filter paper, and dried overnight in hot air oven at 55°C.

### Extraction and assay of PHB

Cyanobacterial biomass was suspended in methanol, kept overnight at 4°C for removal of pigments and then centrifuged at 8000 rpm. The pellet obtained was dried at 60°C. The polymer was extracted in hot chloroform (CHCl_3_) followed by precipitation (with cold diethyl ether) and centrifugation (10,000 g for 20 min). The pellet was washed with acetone, dissolved in hot CHCl_3_ and then transferred in a test tube [12]. The CHCl_3_ was evaporated and conc. H_2_SO_4_ was added. The solution obtained was heated in a boiling water bath. After cooling and thorough mixing, absorbance was measured at 235 nm against H_2_SO_4_ as a blank [13]. Absorbance values were expressed as mg g^-1^ with the help of standard curve of PHB (Sigma-Aldrich, USA).

### Microscopic visualization of PHB

PHB accumulated by *N*. *muscorum* was visualised microscopically after staining with two different dyes, Sudan black B and Nile blue A. For Sudan black B staining, cells were heat fixed onto clean, grease free glass slides and few drops of Sudan black B staining solution (0.3% in 70% ethanol) were added. After 5–10 minutes, the slides were immersed in xylene until complete decolorization (about 10 s), then counterstained with safranine solution (0.5% in water) for 10 s before gently rinsing with running water. The slides were allowed to dry and then examined with an oil immersion lens (Motic, USA) [14].

Nile blue A (1 mg) was dissolved in dimethylsulphoxide (1 ml) to obtain the staining solution. Two drops of the staining solution were added to ~200μl of sterile culture which was then incubated at 55°C for 10 min. Cells were transferred to a glass slide and viewed by a fluorescent microscope (Nikon, USA) at an excitation wavelength of 450–490 nm under 1000× magnification [15].

### Time course study for PHB

The best strain (*N*. *muscorum* NCCU- 442) was cultured in 150ml Erlenmeyer flasks containing 50 ml of BG 11 media and PHB was quantified spectrophotometrically on every 7^th^ for 48 days.

### Optimization for pretreatment solvent/solvent combination, pretreatment technique and extraction process

Five solvents/solvent combinations, i.e. (i) 90% Methanol (ii) 90% acetone (iii) methanol: acetone: water: dimethylformamide [40: 40: 18: 2] (MAD-I), (iv) methanol: acetone: water: dimethylformamide [40: 40: 15: 5] (MAD-II) and (v) methanol: acetone: water: dimethylformamide [40: 40: 10: 10] (MAD-III) were tested for PHB extraction. Impact of shaking (on shaking incubator at 100 rpm) and stirring (with magnetic bar) on PHB extraction was determined as pre-treatment technique.

The potential of eight different solvents, viz. methylene chloride, chloroform, 1.2-dichloromethane, ethyl acetate, dimethylsulphoxide, dimethyforamide, n-hexane and cyclohexane was determined for extraction of PHB using Soxhlet apparatus.

### Optimization of physicochemical culture conditions

The effect of different physical conditions, viz. pH (5–10), temperature (15–45°C) and illumination (continuous light, continuous darkness and light: dark periods [14:10 h]) on PHB yield by *N*. *muscorum* NCCU- 442 was determined.

The impact of chemical conditions, viz. glucose, maltose, fructose, sucrose, lactose and starch as carbon sources (at 0.05%, 0.1%, 0.2% and 0.4%) at three different light and dark periods (14:10 h, 12:12 h & 10:14 h), NaCl addition (0.5, 1.0,1.5, 2.0 gl^-1^) and phosphorus deficiency (K_2_HPO_4_ substituted by equimolar concentration of KCl) was studied for PHB yield.

### FTIR analysis

Potassium bromide pellet was prepared using extracted polymer from *N*. *muscorum* NCCU- 442. Infrared spectra (IR) were recorded using Agilent Cary 630 FT-IR spectrometer with spectral range of 4000–400 cm^−1^ at 27°C.

### ^1^H NMR analysis

The extracted polymer was suspended in deuterochloroform (CDCl_3_) at a concentration of 10 mg ml^–1^. ^1^H NMR spectra were obtained on a Bruker Spectrospin DPX-400MHz NMR spectrometer at 22°C with 7.4 ms pulse width (30° pulse angle), 1 s pulse repetition, 10,330 Hz spectral width, 65,536 data points. Tetramethylsilane was used as an internal shift standard.

### GC-MS analysis

Samples for GC analysis were prepared by propanolysis of the isolated polymer [16]. A mixture of 2 ml 1,2-dichloromethane (DCE) and 2 ml acidified isopropanol (20% v/v hydrochloric acid) was added to polymer at 100°C for 2 h. Phase separation was achieved by adding 4 ml water to the mixture. The propylated 3-hydroxybutyric acid was extracted into the 1, 2-dichloroethane phase and injected directly into the gas chromatograph. The GC-MS analysis was performed with a Shimadzu GC-MS QP 2010 Plus in electron ionization (EI) mode fitted with a RTX-5 (60 m x 0.25 mm x 0.25 μm) capillary column. The carrier gas used was helium with a flow rate of 0.7 ml min^–1^. The injector temperature was 260°C and the initial column temperature of 80°C was held for 2 min before ramping to 250°C at 10°C min^–1^ and holding for 5 min and then finally increased to 280°C at the rate of 15°C min^–1^. A 3.5 min solvent delay was used. Mass spectra were recorded under scan mode in the range of 40–650 m/z. Compounds were identified using NIST11 library.

### TGA analysis

Thermal stability of the extracted polymer was investigated using a Perkin Elmer TGA 4000 under nitrogen atmosphere. 10–15 mg of the extracted polymer was placed in an aluminium pan and then heated from 35 to 700°C with a heating rate of 10°C min^-1^. The characteristic decomposition temperatures viz. initial decomposition temperatures at 2% weight loss (T_onset_), the decomposition temperature at 10% weight loss (T_10_) and temperature of maximum rate of decomposition (T_max_) were determined.

### DSC analysis

DSC analysis was done using a Perkin Elmer DSC 6000 instrument having one sample cell and one reference cell. Approximately 10–15 mg extracted polymer was exposed to a temperature profile over −30°C to 200°C, at a heating rate of 10°C min^-1^ for the first heating scan and then held isothermally for 3 minutes. The sample was cooled to −30°C at -10°C min^-1^ by quenching in liquid nitrogen, and then again reheated to 200°C a heating rate of 10°C min^-1^ during a second heating scan.

The crystallinity (X_c_) of PHB is calculated according to Eq (1).

$$\text{Xc}=\frac{\Delta \text{Hf }\times \;100}{\Delta \text{Ho }}$$ (1)

ΔHf = melting enthalpy of the extracted PHB (J g^-1^), ΔHo = theoretical melting enthalpy of the 100% crystalline PHB which is assumed to be 146.6 J g^-1^ [17]. Indium (m.p. 156.61°C; ΔH = 28.54 J g^-1^) was used for calibrating the DSC apparatus.

### Mechanical properties determination

Polymer film was prepared by conventional solvent casting technique. Powdered polymer was thoroughly dissolved in chloroform to give 15% (w/v) solution under constant stirring on a magnetic stirrer-hotplate at 60 ± 1°C for 20 min. The evaporation of solvent resulted in the formation of PHB film in the glass petri dish. Vacuum drying was done further to remove completely any possible solvent that remained on the film [18]. The tests were done using an Intron Universal Testing Machine Series 4466 (Load Capacity 1kN). The specimens were cut into a rectangular shape and the speed of stretching was 1.0 mm min^-1^. The instrument was connected to a computer, which automatically interpreted the graphical output and computed the tensile strength, Young’s modulus and extension to break ratio. Further, the film surface was observed using a Hitachi S3700 SEM (Scanning Electron Microscope) with an acceleration of 15 kV.

### Biodegradation studies

The biodegradability test used a mixed microbial culture obtained from a soil sample [19]. A nutrient medium was prepared with tryptone (5 g), yeast extract (2.5 g) and NaCl (5 g) in 500 ml of water. 100 g of soil was mixed with 500 ml of water. The suspension was decanted and filtered, and the filtrate was mixed with nutrient media in 1:2 ratio by volume in which PHB and conventional plastic film samples (3cm × 3 cm) were incubated at 28 ± 2°C.

The degradation rate was determined by the ratio of weight loss to the initial weight as shown below:

$$\text{Degradation rate }\left(\text{\%}\right)\;=\;\frac{\left(Wt\;-\;Wo\right)}{Wo}\times 100$$

Where, W_o_ is the initial weight and W_t_ is the weight at test days [20].

### Statistical analysis

The experimental data are presented as mean ± SEM of three replicates. All analysis was conducted using Graph-pad Prism Version-6.0 (Graph Pad Software, San Diego, CA, USA). Statistical analysis of the data was done by one-way analysis of variance (ANOVA).

## Results and Discussion

**Fig 1:**
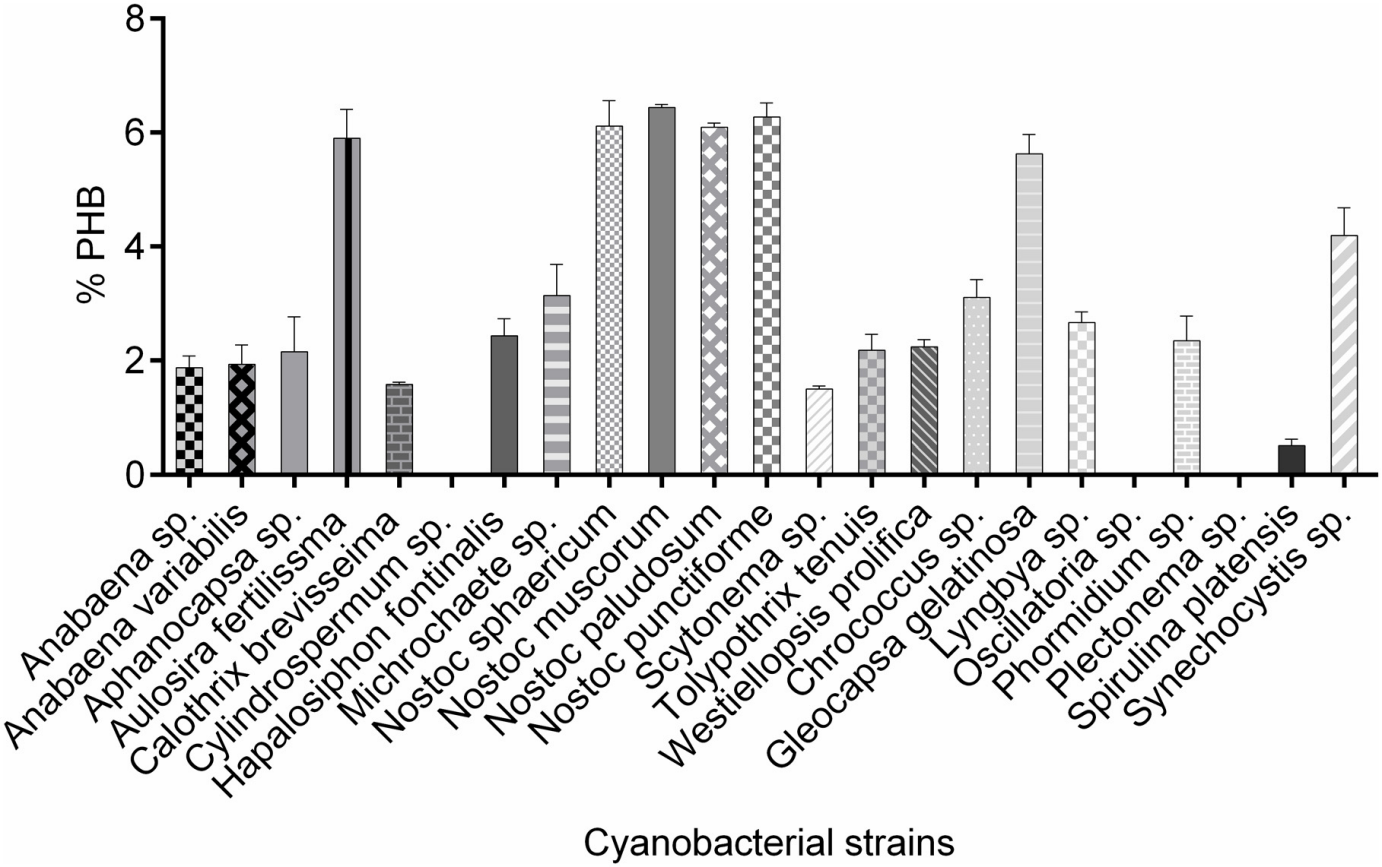
Screening of cyanobacterial strains for PHB (%).

Out of the 23 selected cyanobacterial strains, PHB could be detected in 20 strains and it ranged from 0.51% (*Spirulina platensis*) to 6.44% (*Nostoc muscorum*) (Fig 1). Higher PHB was detected in heterocystous forms, viz. *N*. *muscorum* (6.44%), *N*.*punctiforme* (6.27%), *N*. *paludosum* (6.10%) and *N*. *sphaericum* (6.12%). Slightly higher PHB values were reported in *N*. *muscorum* (8.6%), which could be due to altered culture conditions [21]. *Aulosira fertilissma* came out as the next highest PHB accumulator (5.90%) similar to the PHB value reported earlier [22]. *Michrochaete sp*., *Hapalosiphon fontinalis*, *Westeillopsis prolifica*, *Tolypothrix tenuis* and *Aphanocapsa sp* produced 3.15%, 2.44%, 2.24%, 2.18% and 2.16% PHB, respectively. A relatively higher PHB value was recorded earlier for *Aphanocapsa sp*. (3.02%) and lower values for *W*.*prolifica* (1.63%) and *T*. *tenuis* (1.96%) [21]. *Anabaena variabilis* and *Anabaena sp*. produced 1.94% and 1.87% PHB, respectively. *Calothrix brevissima* and *Scytonema sp*. showed 1.58% and 1.50% PHB. No PHB accumulation was noticed in *Cylindrospermum sp*., which is in agreement with the earlier work of Mallick [21].

Among the non heterocystous forms, *Gleocapsa gelatinosa* (5.63%) stood out as the best PHB producer. It was followed by *Synechocystis sp*. (4.20%) and *Chrococcus sp*. (3.11%). Earlier reports showed 4.5% PHB in *Synechocystis sp*.PCC6803 harvested at stationary phase under normal conditions [23]. *Lynbgya sp*. and *Phormidium sp*. produced 2.66% and 2.35% PHB respectively. *Plectonema* and *Oscillatoria sp*. came out as the non-accumulators of PHB among the non-N_2_ fixing cyanobacteria. The negative results for *Plectonema* substantiate the findings of Mallick [21]. *Spirulina platensis* accumulated 0.51% PHB. Similar amount of PHB (0.7%, 0.8% and 0.4%) was also detected in *S*.*platensis* LB1475/4, *S*.*platensis* M13 and *S*.*maxima* SOSA 18 respectively [24].

**Fig 2:**
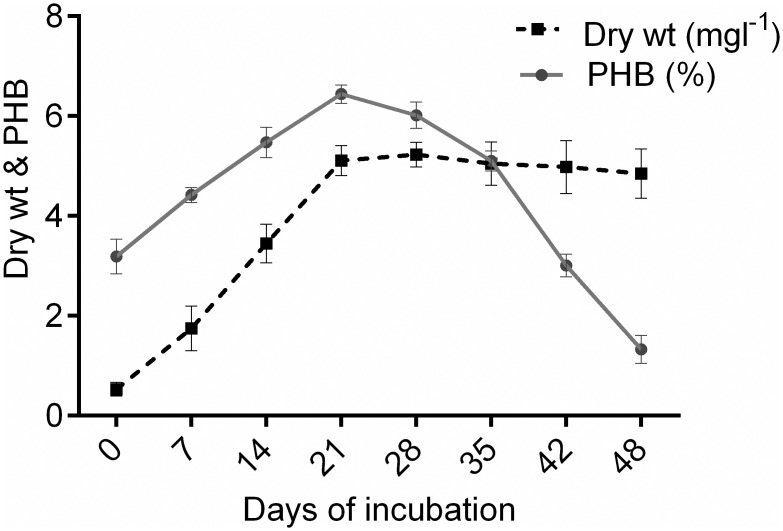
Time course study of *Nostoc muscorum* NCCU- 442 with respect to PHB (%) and growth (dry weight).

The highest PHB-yielding strain, *Nostoc muscorum* NCCU- 442, was selected for further experiments (time course study, optimization of extraction process, culture conditions, characterization, determination of mechanical and biodegradation properties). During time course, PHB accumulation started in the early phase of growth, but the maximum accumulation was recorded on 21^st^ day (6.44%) with significance increase (p < 0.0001) as compared with the initial day (Fig 2). Earlier workers also found the maximum PHB accumulation in *Nostoc muscorum* (8.6%) and *Synechocystis sp*. (4.55%) respectively on the 21^st^ day [21,23]. A decrease in PHB after 21 days may be due to its utilization for cell functioning of *N*. *muscorum* NCCU- 442.

**Fig 3:**
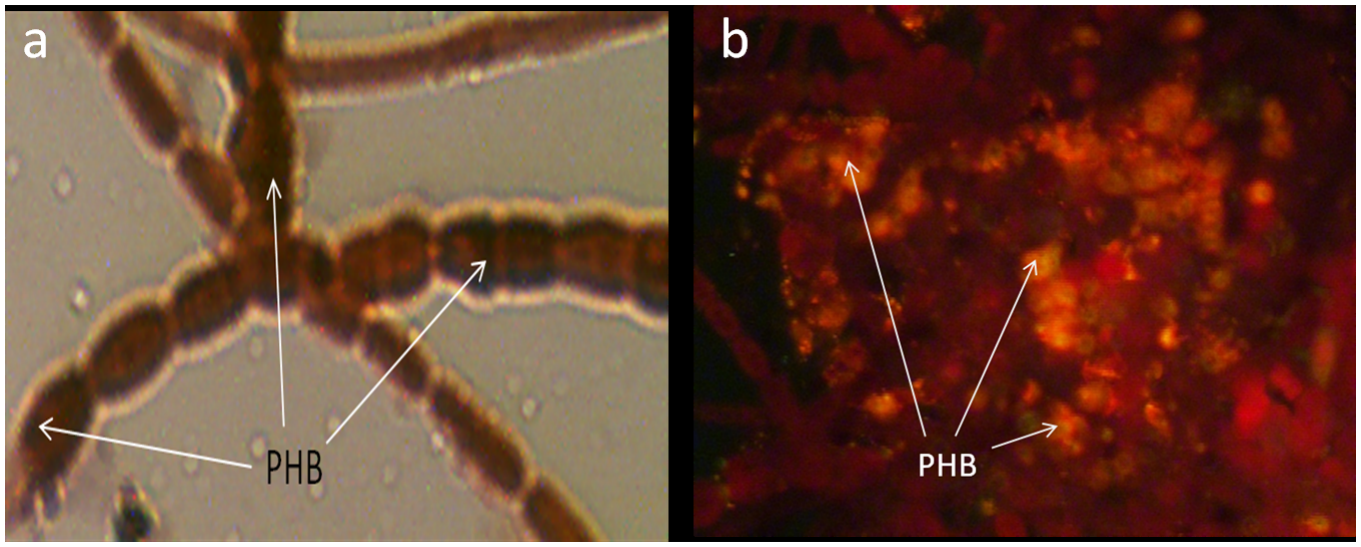
Microscopic visualization of *Nostoc muscorum* NCCU- 442 for PHB. (a) With Sudan black B (b) With Nile blue A.

During the microscopic visualization, PHB appeared as black granules with Sudan black B (Fig 3a) as observed by other workers [14], whereas they appeared as brightly fluorescent orange granules with Nile blue A (Fig 3b) staining similar to the previous studies [15].

**Fig 4:**
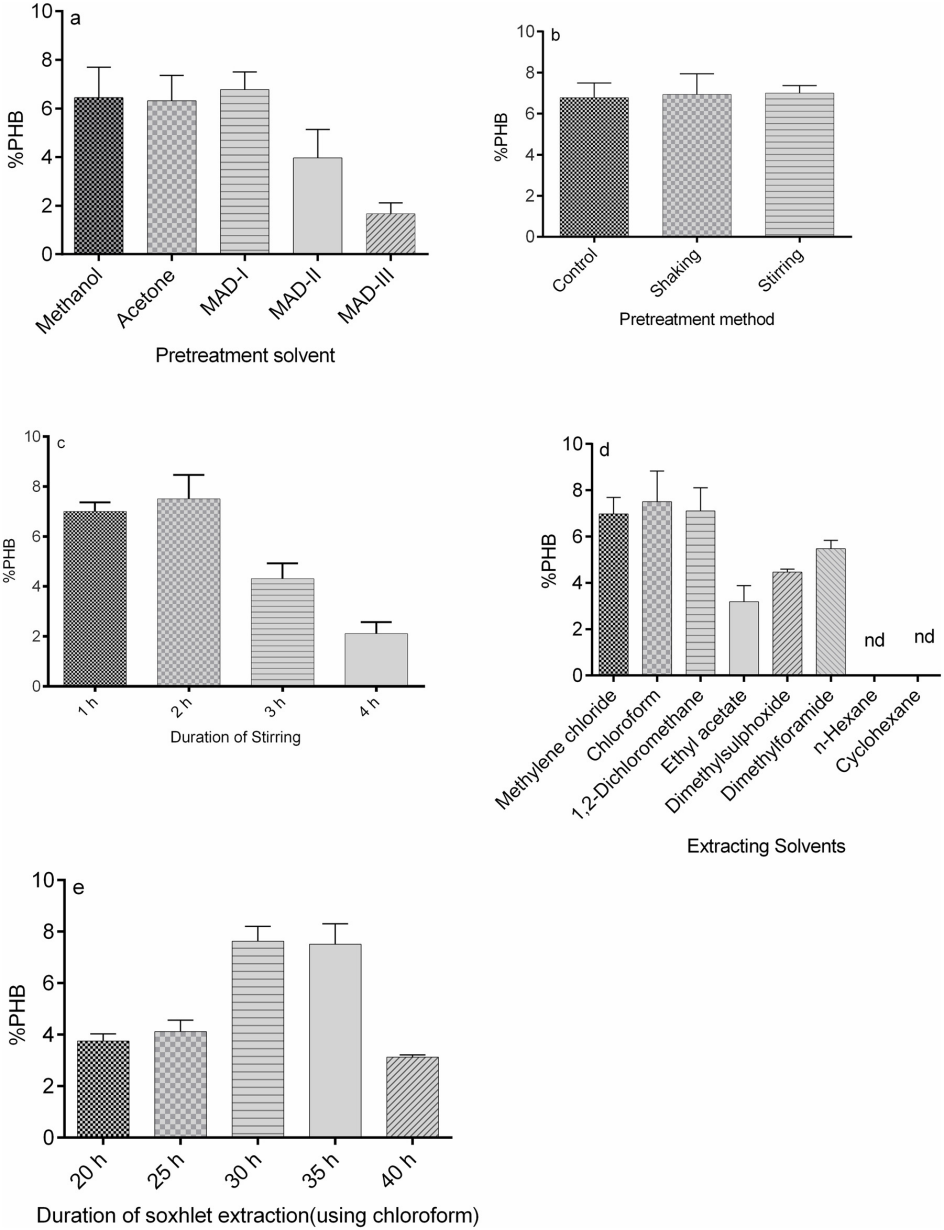
(a-e). Optimization of PHB extraction process in *Nostoc muscorum* NCCU- 442. (a). Effect of addition of pre-treatment solvents, (b) shaking/ stirring during pre-treatment, (c) duration of pre-treatment, (d) solvent nature during soxhlet extraction (e) Duration of soxhlet extraction (using chloroform).

The present study used five solvent/solvent combinations: methanol, acetone, MAD-I, MAD-II and MAD-III. Highest PHB (6.78%) was obtained with MAD-I (Fig 4a) which may be due to the presence of 2% DMF. DMF, being a good pigment extractor, especially for resistant cyanobacteria and coccoid algae, removed the pigments in the pretreatment step, which ultimately facilitated the easy diffusion of PHB during soxhlet extraction [25,26]. Methanol and acetone resulted in 6.44% and 6.32% PHB respectively. Decrease in PHB yield in MAD-II (3.96%) and MAD-III (1.66%) may be due to a higher DMF (5% and 10% respectively) that may have removed PHB also along with the pigments since DMF is a good solvent of PHB as well [27].

Stirring (with magnetic bar) and continuous mechanical shaking (at 100 rpm) of the cultures for 1 h as pre-treatment technique resulted in 7% and 6.90% PHB respectively (Fig 4b), while in the control (without any shaking/ stirring), PHB yield was 6.78%. The slight increase in PHB yield with stirring may be due to better diffusion of pigments from the cyanobacterial cell into the pre-treatment solvent, which indirectly affects the PHB extraction. In an attempt to increase the PHB yield further, duration of stirring with the magnetic bar was raised from 1 h to 4 h. The highest PHB (7.50%) was obtained with 2 h stirring, while further increase in stirring (3 h and 4 h) decreased the PHB yield (Fig 4c).

In the present study, selection of the solvent for continuous soxhlet extraction and the duration of contact time with the solvent were also optimised. PHB extraction was the highest with chloroform (7.50%) and it was followed by 1, 2-dichloroethane (7.11%), methylene chloride (6.98%), dimethylforamide (5.48%) and dimethylsulphoxide (4.46%) (Fig 4d). The lowest PHB extraction occurred with ethyl acetate (3.19%) due to partial solubility of PHB. Insolubility of PHB in n-hexane and cyclohexane may be the reason for its non-detectability in these solvents [27].

Duration of soxhlet extraction with the best extracting solvent (chloroform) for 20, 25, 30, 35 & 40 h showed remarkable impact on PHB extraction from *N*. *muscorum* NCCU- 442. The maximum PHB (7.63%) was noticed after 30 h (Fig 4e).

Thus, it was observed that PHB yield increased upto 7.63% with the new developed extraction protocol in comparison to 6.44% as obtained with the conventional protocol i.e. a substantial 18.47% increase in PHB yield was achieved.

**Fig 5:**
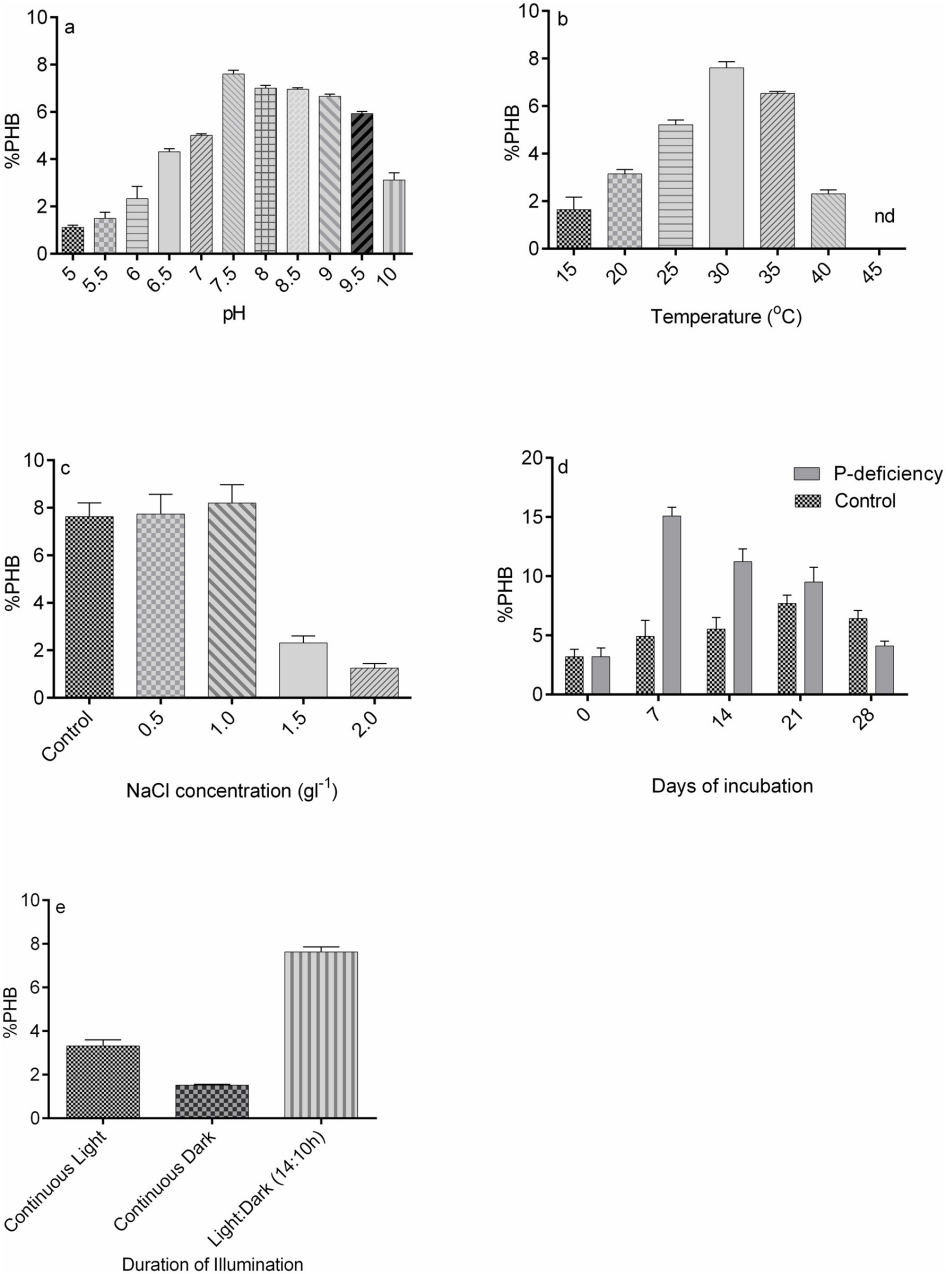
(a-e). Optimization of culture conditions of *Nostoc muscorum* NCCU- 442 for PHB yield. (a) Effect of pH, (b) temperature, (c) NaCl-addition, (d) P- deficiency (e) Duration of light and dark periods.

An alkaline pH (8–9.5) favoured more PHB accumulation than acidic pH (5–7) (Fig 5a). The maximum PHB (7.60%) accumulation occurred at pH 7.5. High PHB accumulation is reported in *Spirulina platensis* at pH 9.0 [28] and in *Synechocystis sp*. PCC6803 at pH 8.5 [23].

During temperature optimization, 30°C yielded the maximum PHB (7.61%) with a significant increase (P < 0.0001), as compared to 15, 20, 25, 40, 45°C (Fig 5b). The maximum PHB is reported at 28°C in cyanobacterium *Synechocystis sp*.PCC6803 [23]. Same temperature (30°C) was observed as an optimal temperature for PHB accumulation in four bacterial species, viz. *Azohydromonas lata* DSMZ 1123, *Hydrogenophaga pseudoflava* DSMZ 1034, *Cupriavidus necator* DSMZ 545 and *Azotobacter beijinckii* DSMZ 1041[29].

Under salt stress, *N*. *muscorum* NCCU- 442 showed slight increase in PHB accumulation (7.74% and 8.15% at 0.5 and 1.0 gl^-1^ NaCl respectively), as compared to control (7.63%) at the 7^th^ day (Fig 5c). It was suggested that by storing large quantities of reduced carbon in the form of PHB during osmotic stress, salt tolerant bacterial cells might balance the osmotic pressure imposed by the environment [30]. Since *N*. *muscorum* NCCU- 442 is a freshwater strain, PHB decreased with further increase in NaCl concentrations (1.5 and 2.0 g l^-1^). A direct effect of NaCl concentration on citrate synthase in the TCA cycle results in an increased acetyl CoA pool that favours PHB synthesis in the marine cyanobacterium *Spirulina subsalsa* [15]. At the same time, this decreases the free CoA concentration which reduces the inhibition of 3-ketothiolase, which catalyses the acetoacetyl-CoA formation from two molecules of acetyl CoA during PHB synthesis.

Phosphorus deficiency enhanced PHB accumulation (15%) on 7^th^ day, as compared to the control (4.9%) (Fig 5d). Increased PHB accumulation during phosphate limitation has also been observed in *Synechococcus sp*. MA19 [31]. ATP production decreases during phosphate limitation whereas reduction of NADP yielding NADPH through noncyclic photosynthetic electron flow continues, resulting in an increased NADPH pool that is utilised in PHB synthesis [32]. Also, the high levels of NADPH and NADH inhibit citrate synthase in the TCA cycle [33], which in turn leads to increased PHB accumulation. However, a prolonged phosphate deficiency limits the major physiological processes such as nucleic acid synthesis, protein synthesis etc. which indirectly stops PHB synthesis.

Cells grown in 14:10 h light-dark photoperiod were found to be better PHB accumulators (7.63%) than those grown in continuous illumination (3.31%) or continuous darkness (1.51%) (Fig 5e). Greater PHB accumulation in cultures grown in light-dark cycles than those grown under continuous light agrees with the findings of other scientists [34], which hypothesized that dark periods are obligatory for PHB accumulation in phototrophic cultures. A higher PHB accumulation (3.31%) under continuous illumination than in complete darkness (1.51%) suggested a light-dependent PHB synthesis in *N*. *muscorum* NCCU-442. Higher PHB accumulation in continuous illumination than complete darkness was also observed in photoautotrophic *Spirulina platensis* [28].

For the normal physiological functioning of cells, four fundamental elements, viz. C, H, O and N, are needed. Water is the most common source of H and O. However, microorganisms obtain C and N from different sources depending on their mode of nutrition. Under low light intensity, interrupted illumination or complete darkness, exogenous organic carbon is required for growth. Carbon sources are utilised by the normal cyanobacterial cell for biomass synthesis, cell maintenance and the excess C after fulfilling the above processes is used in PHB synthesis, especially in stress conditions [35]. Effect of duration of photoperiod was also included in carbon source-preference studies, as the heterotrophic growth of cyanobacteria is dependent mainly on the dark period. In the present study, all the carbon sources, especially glucose, supported significantly (P < 0.0001) higher PHB accumulation in *N*. *muscorum* NCCU- 442 than photoautotrophy (Table 1). PHB accumulation was 18.01%, 20.52% and 24.14% at 0.4% glucose concentration in 14:10, 12:12 and 10:14 h light:dark periods, respectively. Some species of *Nostoc*, including *Nostoc muscorum*, grow heterotropically on monosccharides like glucose, disaccharides (maltose, sucrose) under dark conditions [36–38]. The oxidative pentose phosphate (OPP) pathway is the major route of catabolism of endogenous glycogen for dark survival or of exogenously supplied carbohydrates that provide biosynthetic intermediates [39]. The pathway increases the reducing cofactor NADPH pool which is required for activity of the enzyme acetoacetyl CoA reductase of the PHB-biosynthetic-pathway [40].

**Table 1:** Effect of carbon supplementation and light and dark periods on PHB (% w/w of dry cells) in *Nostoc muscorum* NCCU-442.

**Carbon supplementation under 14:10 h (light:dark) period**					
**Source**	**Concentration (%)**				
	**Control**^#^	**0.05%**	**0.1%**	**0.2%**	**0.4%**
Glucose	7.63±0.46	9.11±1.17	13.67±1.06	16.52±0.80	18.01±1.31
Lactose	7.63±0.46	7.64±0.46	7.69±1.23	7.73±0.55	8.04±1.19
Sucrose	7.63±0.46	7.73±0.66	8.66±0.24	9.38±1.09	10.15±0.62
Fructose	7.63±0.46	7.92±0.78	8.66±1.06	9.46±0.89	10.32±0.71
Maltose	7.63±0.46	7.64±0.92	7.77±1.30	8.00±0.83	8.07±0.83
Starch	7.63±0.46	7.64±0.55	7.76±1.15	7.86±1.03	7.98±0.79
**Carbon supplementation under 12:12 h (light:dark) period**					
**Source**	**Concentration (%)**				
	**Control**^#^	**0.05%**	**0.1%**	**0.2%**	**0.4%**
Glucose	7.61±0.32	9.54±1.04	15.32±0.91	17.41±0.70	20.52±0.88
Lactose	7.61±0.32	7.69±0.86	7.81±0.66	8.00±0.65	8.12±0.54
Sucrose	7.61±0.32	8.00±0.79	9.09±1.44	9.78±0.97	11.46±0.52
Fructose	7.61±0.32	8.10±0.73	9.31±1.33	10.01±0.83	11.81±0.83
Maltose	7.61±0.32	7.67±0.99	7.92±0.79	8.00±0.37	8.22±0.98
Starch	7.61±0.32	7.68±0.68	7.80±0.66	7.91±0.08	8.00±0.07
**Carbon supplementation under 10:14 h (light:dark) period**					
**Source**	**Concentration (%)**				
	**Control**^#^	**0.05%**	**0.1%**	**0.2%**	**0.4%**
Glucose	7.60±1.02	10.11±0.86	17.61±1.12	20.12±1.34	24.14±0.95
Lactose	7.60±1.02	7.69±0.60	7.91±1.29	8.01±1.18	8.22±0.96
Sucrose	7.60±1.02	8.37±0.72	9.19±0.45	10.31±0.93	12.35±1.09
Fructose	7.60±1.02	8.54±0.43	9.38±0.92	10.75±0.69	13.67±0.94
Maltose	7.60±1.02	7.73±0.06	7.98±0.61	8.05±1.12	8.40±0.87
Starch	7.60±1.02	7.70±0.54	7.84±0.66	7.93±0.99	8.04±0.10

^#^ Under photoautotrophy. Data are given as means value ± SEM of three replicates (n = 3).

The cumulative effect of all the optimised conditions on PHB yield in *N*. *muscorum* NCCU- 442 was determined. The *N*. *muscorum* NCCU- 442 yielded 4.42% PHB under normal conditions on 7^th^ day while at the same day, about six times more PHB, amounting to 26.37% was detected under optimized conditions. Previous studies with *Nostoc muscorum* Agardh reported up to 40–43% PHB under gas-exchange limitations supplemented with 0.4% (w/v) acetate, after 21 days culturing under usual light:dark cycles (14:10 h) and further 5–7 days dark incubation [41]. In other words, a total of 26–28 days were required to produce 40–43% PHB. But within this time period, our study can used to produce significant PHB upto 105.48% i.e. about 2.4–2.6 times more PHB than the above study.

**Fig 6:**
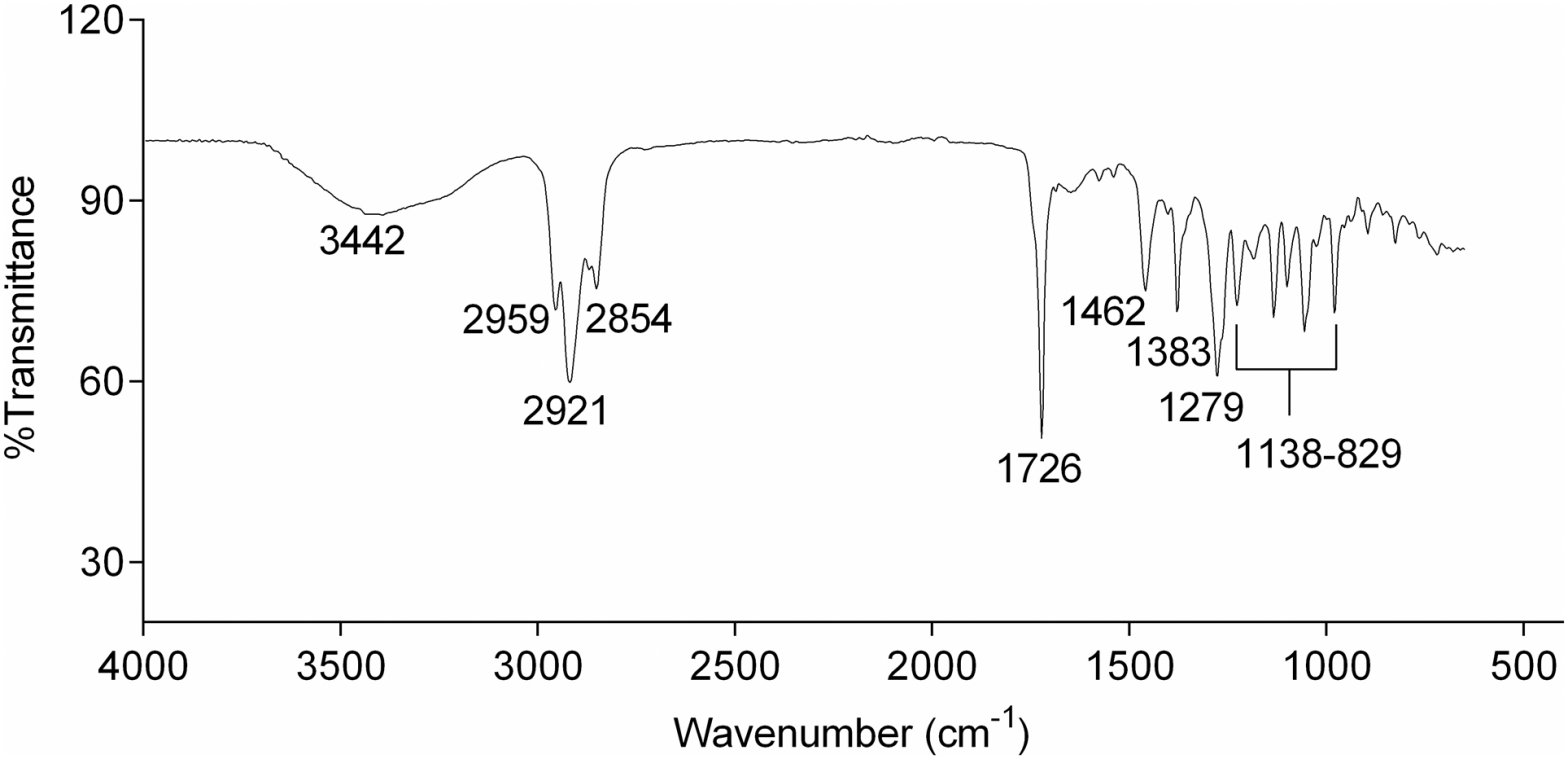
FTIR spectrum of isolated PHB from *Nostoc muscorum* NCCU- 442.

During characterization of isolated PHB, the FTIR spectrum showed prominent peaks at 1726 cm^-1^ and 1279 cm^-1^ (Fig 6). These peaks denote carbonyl (C = O) and asymmetric C-O-C stretching vibration, respectively, characteristic for ester bonding found in PHB molecule. Other adsorption bands obtained at 1383, 1462, 2959–2854, and 3442 cm^-1^ denote the -CH_3_, -CH_2_, -CH, and O-H groups respectively. The absorption bands at 1138 cm^-1^ to 829 cm^-1^ were consigned to C-O and C-C stretching vibration which could be attained by amorphous PHB [42]. Almost identical peaks at 1382, 1726, 2978, 2934, 3439 cm^-1^, denoting the various functional groups of PHB marine cyanobacterium, *Spirulina subsalsa* were observed [15].

**Fig 7:**
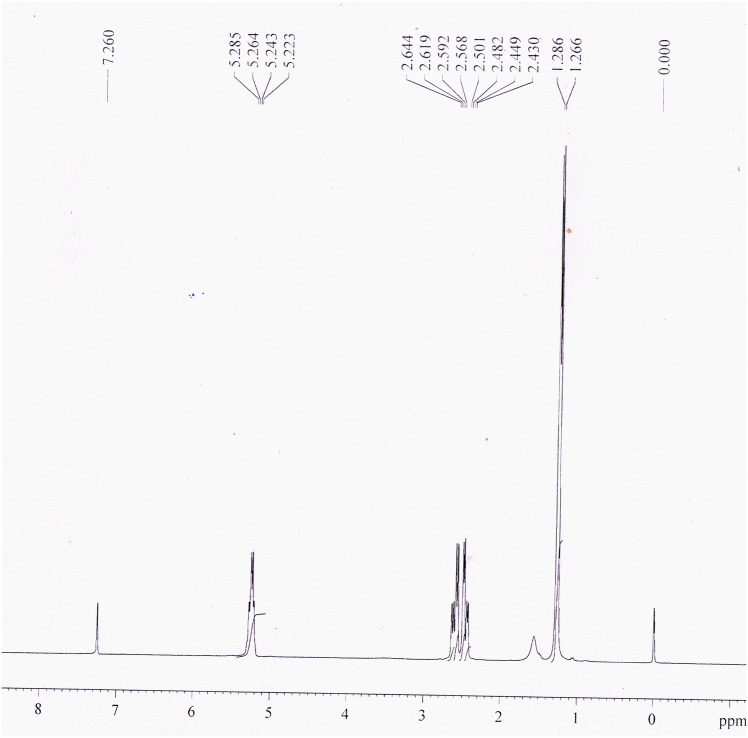
^1^H NMR spectrum of isolated PHB from *Nostoc muscorum* NCCU- 442.

^1^H NMR spectrum of the isolated polymer dissolved in deuterochloroform showed a multiplet between 5.22 and 5.28 ppm denoting the methine proton (-CH) having the chiral carbon (Fig 7). Diastereotopic methylene (-CH_2_) protons were assigned to the double quadruplet having the resonance value 2.43–2.50 ppm and 2.57–2.64 ppm. Finally, the methyl protons (-CH_3_) gave doublet signals at 1.27–1.29 ppm. These chemical shift signals with respect to internal standard tetramethylsilane (peak at 0 ppm) are almost identical with the chemical shift signals obtained for PHB produced by *Cupriavidus necator* [43].

**Fig 8:**
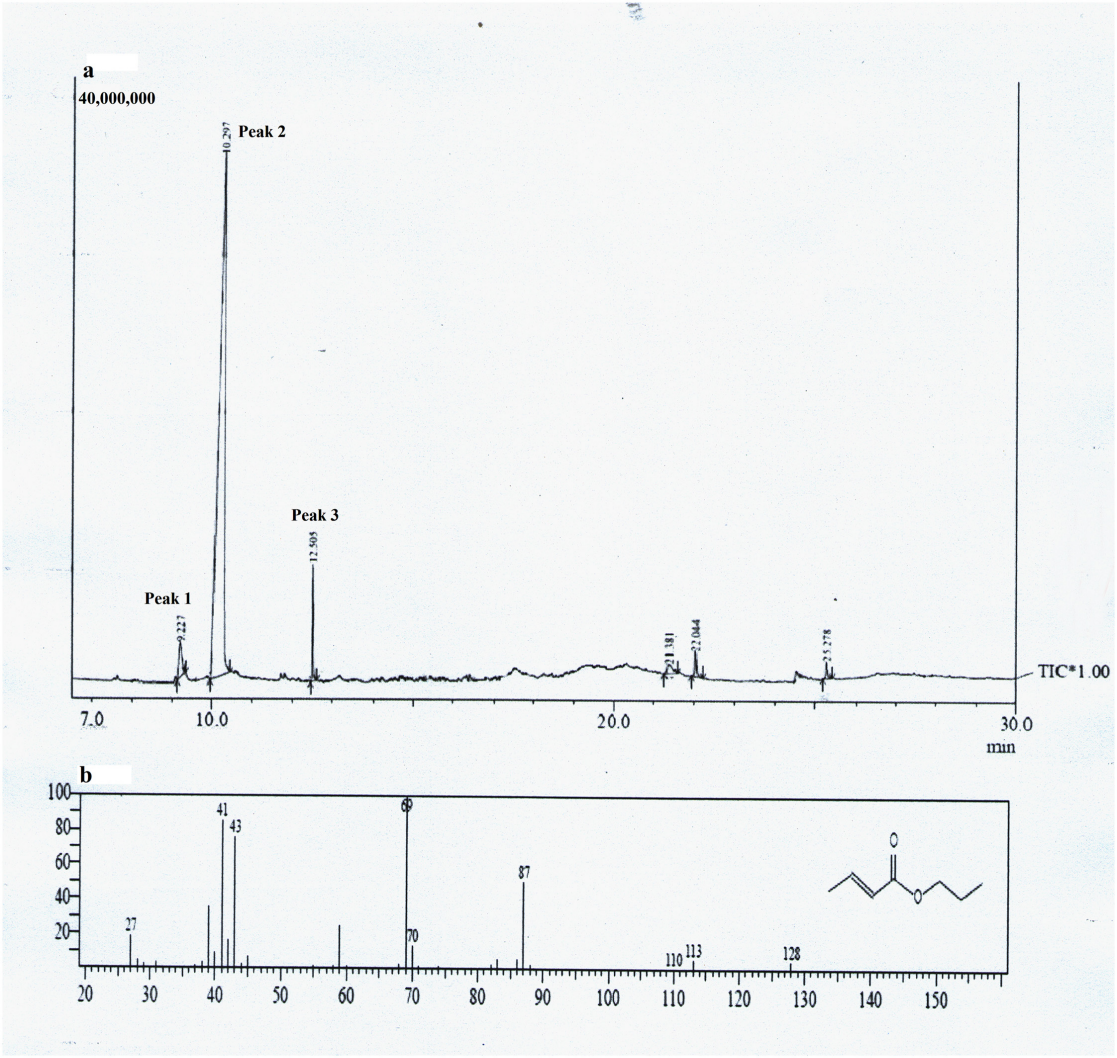
GC MS analysis. (a) GC spectra of isolated PHB from *Nostoc muscorum* NCCU- 442 (b) Comparision of the peak (Rt-10.297) with mass spectra MS library (NIST 11).

The gas chromatogram of the isolated polymer showed a major peak with retention time 10.297 min (Peak 2) with two minor peaks at 9.227 (Peak 1) and 12.505 min (Peak 3) (Fig 8a). As identified by comparing molecules in the GC database, the major peak represents isopropyl ester of 2-butenoic acid confirming the polymer as PHB (Fig 8b). The first minor peak at 9.227 min represents the solvents used during sample preparation, while the second minor peak at 12.505 denotes benzoic acid isopropyl ester. Same sequence of these components as represented by the 3 peaks in our study was also obtained by earlier workers [16]. The area under the major peak denoted the PHB content in dry cell mass and it was determined to be 88.25%.

**Fig 9:**
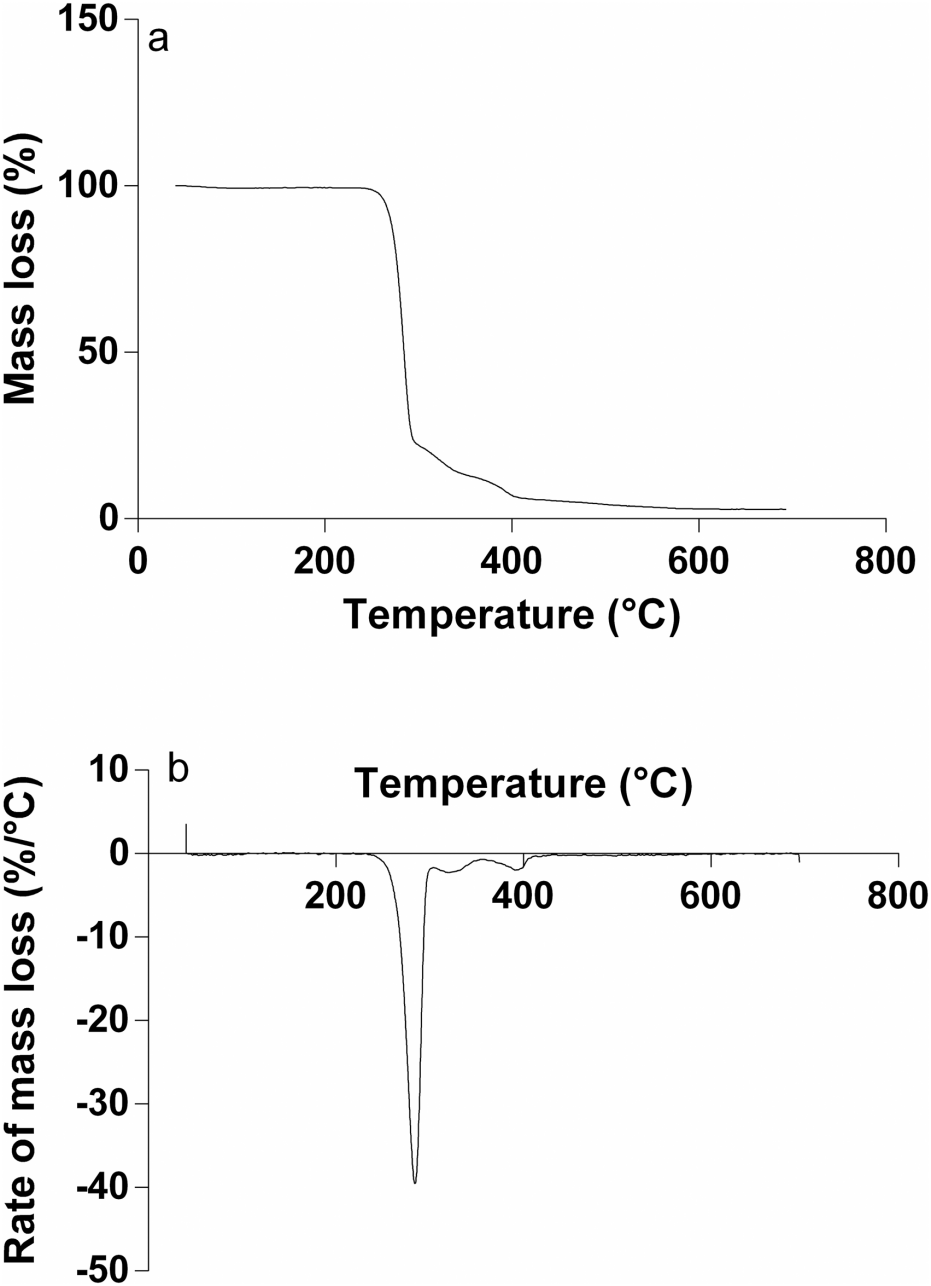
TGA analysis. (a) TGA thermogram and (b) DTG curve of isolated PHB from *Nostoc muscorum* NCCU- 442.

The thermal degradation of PHB is known to occur through random chain scission reaction of the PHB ester groups to form shorter chains with carboxylic and olefinic terminal groups [44]. The characteristic decomposition temperatures viz. initial decomposition temperatures at 2% weight loss (T_onset_) and the decomposition temperature at 10% weight loss (T_10_) were determined directly from the TGA curve (Fig 9a) whereas the temperature of maximum rate of decomposition (T_max_) was determined with the help of the respective derivative thermogravimetric (DTG) curve of the extracted polymer (Fig 9b). The T_onset_, T_10_ and T_max_ were determined to be 256°C, 270°C and 284°C respectively. T_onset_ value (but determined for 5% weight loss) and T_max_ for standard PHB (KOHAP Ltd. Korea) had been found to be 267°C and 295°C respectively [45]. Another study determined T_10_ value to be 260°C for standard PHB from Sigma [15].

**Fig 10:**
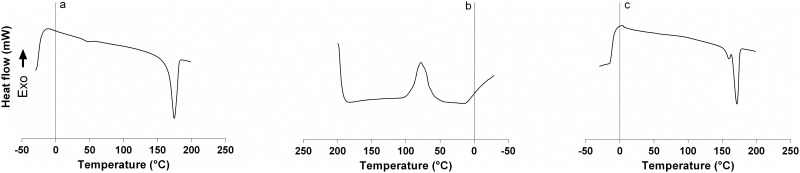
DSC analysis of isolated PHB from *Nostoc muscorum* NCCU- 442. (a) during first heating scan (b) Cooling scan (c) Second heating scan.

The DSC thermograms were used to determine the characteristic calorimetric parameters viz. melting temperature (T_m_), glass transition temperature (T_g_) and crystallization temperature (T_c_) of the extracted polymer. The first heating scan (-30°C to 200°C) was used to erase any prior thermal history of the sample (Fig 10a). T_c_ was determined to be 78.8°C as the peak value of the DSC curve obtained during the cooling scan from 200°C to -30°C (Fig 10b). During the second heating scan from -30°C to 200°C, the T_g_ and T_m_ were calculated directly as the inflection point and the peak value respectively of the obtained DSC curve (Fig 10c). The respective values determined were 6°C and 171°C. The melting enthalpy determined during this scan was used to calculate the crystallinity of the PHB following the Eq 1. High melting enthalpy of about 83.25 J g^-1^ suggested the highly crystalline nature of the extracted polymer which was calculated to be 56.78. Similar values of melting temperature (177°C), glass transition temperature (9°C), crystallization temperature (67°C) and melting enthalpy (80 J g^-1^) had been determined for standard PHB (KOHAP Ltd. Korea) previously [45].

PHB film (S1a Fig) produced by solvent casting technique was transparent and brittle. During scanning electron microscopy (SEM), it showed no void spaces or agglomeration that suggested homogenous surface morphology (S1b Fig) and uniform dispersion PHB in chloroform.

**Fig 11:**
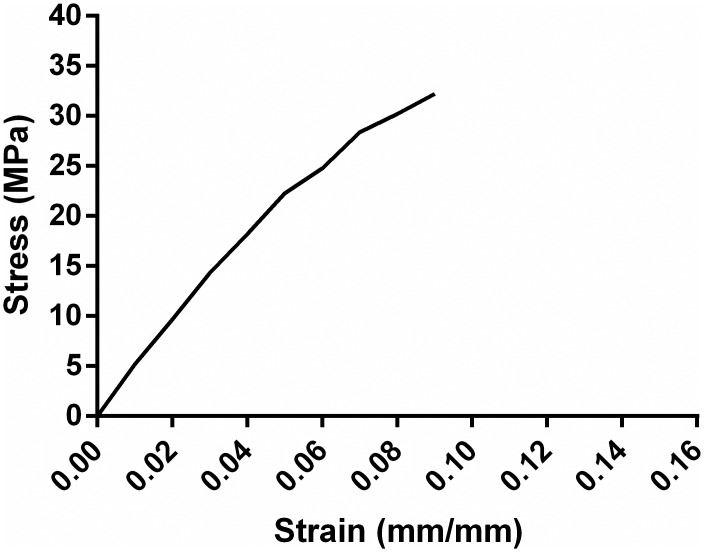
A typical stress-strain curve of PHB film.

Fig 11 depicts a typical stress-strain curve obtained during mechanical testing of rectangular pieces of *N*. *muscorum* NCCU- 442 derived PHB film. Three mechanical properties viz. tensile strength, extension to break ratio and Young’s modulus were determined for the PHB film. Tensile strength is the stress (force) required to rupture a sample whereas elongation to break is the strain (deformation) produced on a sample when it ruptures. Young's modulus is the ratio of stress to strain and is the slope of the stress-strain curve [46]. The quantitative values for the three mechanical properties were calculated to be 31.1 MPa, 8.6% and 1.5 GPa, respectively. Tensile strength and extension to break ratio of the standard PHB (Serrana, SP, Brazil) were calculated to be 28 MPa and 9% respectively [47]. Youngs modulus value of standard PHB was also reported to be 1.7 GPa [48].

**Fig 12:**
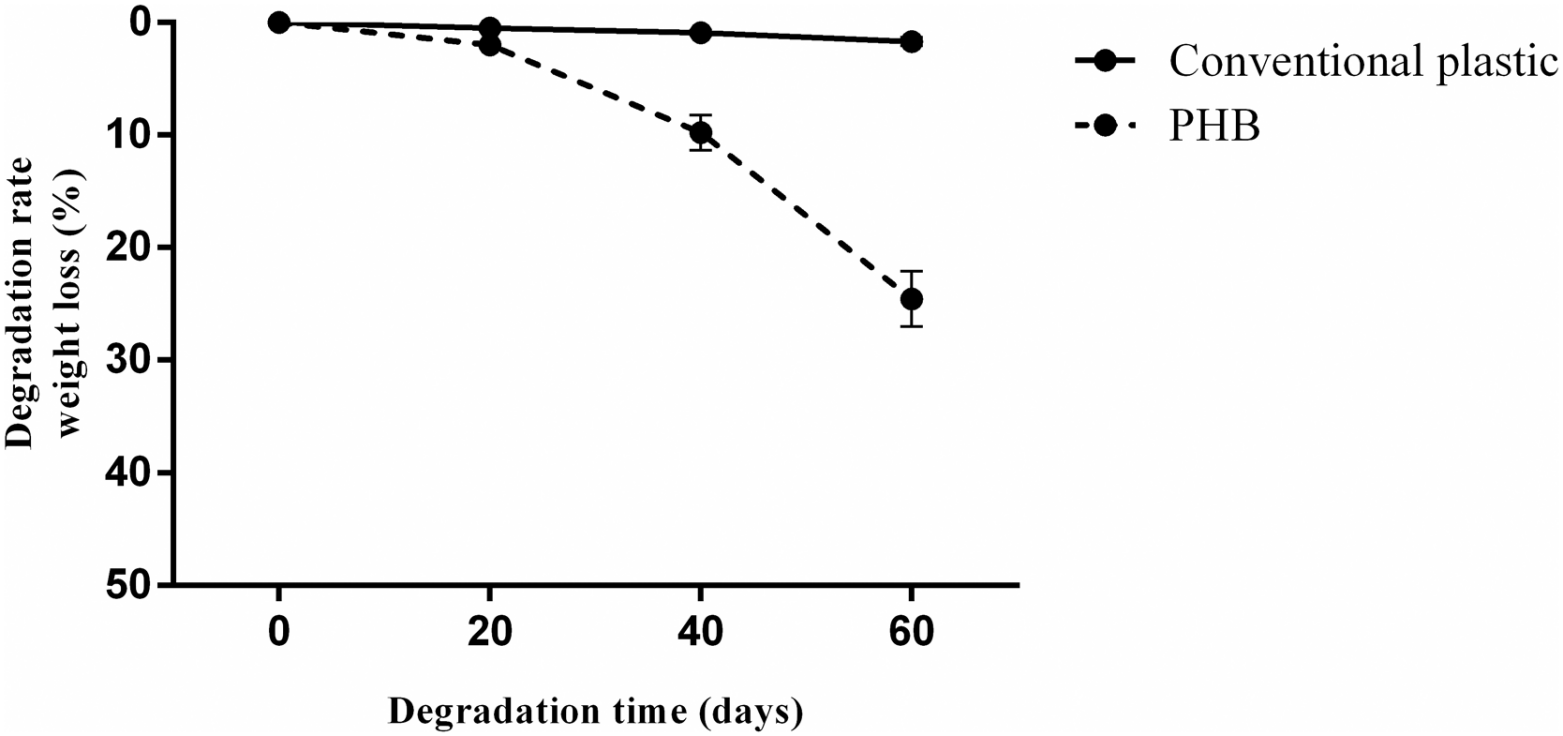
Biodegradation potentiality of conventional plastic and PHB film with mixed microbial culture.

Environmental friendly nature of the *N*. *muscorum* NCCU- 442 derived PHB film was verified by its degradation potential (as weight loss ratio) in presence of mixed microbial soil culture. After 60 days, square shaped cut PHB film was degraded but the conventional plastic was almost unaffected (S2 Fig). The weight loss of PHB film and conventional petrochemical plastic were 24.58% and 1.68% respectively (Fig 12). Earlier, food packaging products made of standard PHB (Serrana, SP, Brazil) have been shown to undergo effective biodegradation in different environments (potable water, aviary bed, garden soil etc.) for periods of up to 60 days [49]. In fact, various aerobic and anaerobic PHB-degrading microorganisms have been studied [50].

## Conclusion

The majority of the cyanobacterial strains in the present study showed PHB accumulation, proving cyanobacteria as a good source of PHB. A new optimised extraction protocol for PHB extraction from cyanobacteria, which includes pre-treatment of biomass with MAD-I with 2 h magnetic bar stirring and continuous 30 h chloroform soxhlet extraction was developed. The developed extraction protocol resulted in 18.47% increase in PHB yield as compared to the conventional extraction protocol. The optimum physicochemical conditions for PHB accumulation were: pH 7.5, 30°C, 10:14 light and dark periods with 0.4% glucose (as additional C source), P-deficiency and 1.0 gl^-1^ NaCl. Under these optimised conditions, *Nostoc muscorum* NCCU- 442 accumulated 26.37% PHB, as compared to 4.42% (during controlled conditions) on 7^th^ day. FTIR, NMR and GC MS analyses confirmed the isolated polymer as PHB. Thermal properties and mechanical properties of the isolated PHB were similar to standard PHB. Biodegradation test showed that cyanobacterial PHB degrade in lesser time and thus can act as environmental-friendly substitute for petroleum-based plastics. Hence, it may be concluded that *N*. *muscorum* NCCU- 442 can be exploited for PHB production at a large scale.

## Supporting Information

S1 Fig Morphological studies of PHB film. (a) Through simple camera (b) Scanning Electron Microscope (SEM).
(TIF)

S2 Fig Biodegradation test. (a) Photograph of the conventional plastic (CP) and PHB film at zero time and (b) at 60^th^ day.
(TIF)

## Abbreviations

PHB: Polyhydroxybutyrate
FTIR: Fourier Transform Infrared spectroscopy
^1^H NMR: proton Nuclear Magnetic Resonance
GC-MS: Gas Chromatography- Mass Spectrometry
TGA: Thermogravimetric analysis
DSC: Differential Scanning Calorimetry
NaCl: Sodium chloride
CoA: Co-enzyme A
