# Supplementary figures and images for: Cyanobacterial Polyhydroxybutyrate (PHB): Screening, Optimization and Characterization
Source: PLoS One. 2016 Jun 30;11(6):e0158168. doi: 10.1371/journal.pone.0158168 (PMC4928839; doi:10.1371/journal.pone.0158168)

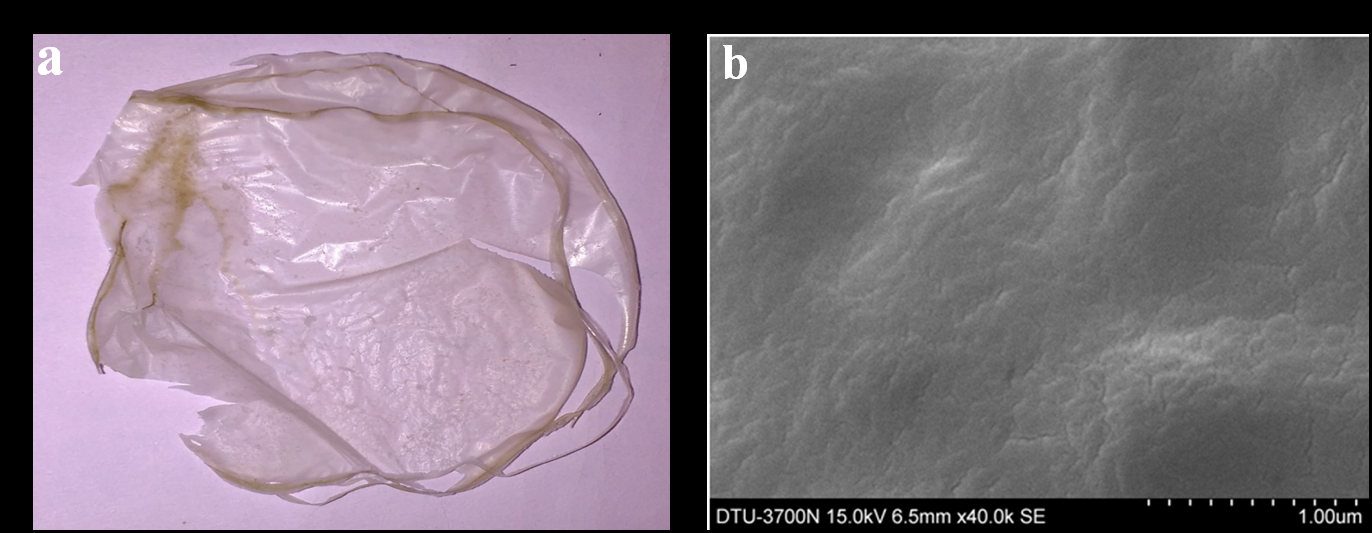

Supplement: S1 Fig — (a) Through simple camera (b) Scanning Electron Microscope (SEM). (TIF) [file pone.0158168.s001.tif]

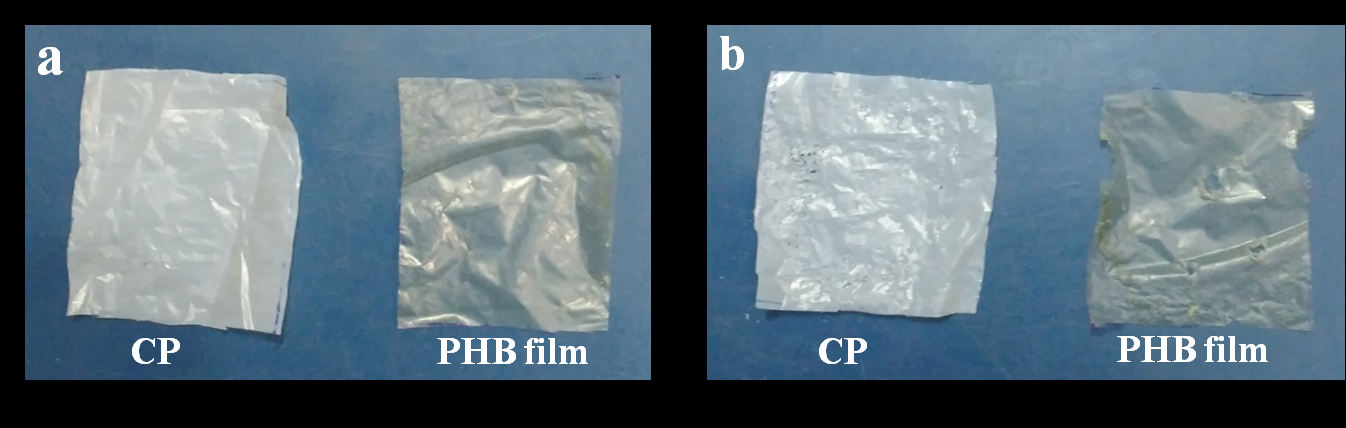

Supplement: S2 Fig — (a) Photograph of the conventional plastic (CP) and PHB film at zero time and (b) at 60th day. (TIF) [file pone.0158168.s002.tif]
